# Andrographolide Sulfonate Is a Promising Treatment to Combat Methicillin-resistant *Staphylococcus aureus* and Its Biofilms

**DOI:** 10.3389/fphar.2021.720685

**Published:** 2021-09-16

**Authors:** Lulu Zhang, Bo Wen, Mei Bao, Yungchi Cheng, Tariq Mahmood, Weifeng Yang, Qing Chen, Lang Lv, Li Li, Jianfeng Yi, Ning Xie, Cheng Lu, Yong Tan

**Affiliations:** ^1^ Institute of Basic Research in Clinical Medicine, China Academy of Chinese Medical Sciences, Beijing, China; ^2^ Key Laboratory for Research on Active Ingredients in Natural Medicine of Jiangxi Province, Yichun University, Yichun, China; ^3^ Department of Pharmacology, Yale University School of Medicine, New Haven, CT, United States; ^4^ Department of Plant Sciences, Faculty of Biological Sciences, Quaid-i-Azam University, Islamabad, Pakistan; ^5^ Medical Experimental Center, China Academy of Chinese Medical Sciences, Beijing, China; ^6^ Qingfeng Pharmaceutical Co. Ltd., Ganzhou, China

**Keywords:** andrographolide sulfonate, methicillin-resistant *Staphylococcus aureus*, drug-resistant biofilm, RT-PCR, metabonomics

## Abstract

Methicillin-resistant *Staphylococcus aureus* (MRSA) is a drug-resistant pathogen threatening human health and safety. Biofilms are an important cause of its drug resistance and pathogenicity. Inhibition and elimination of biofilms is an important strategy for the treatment of MRSA infection. Andrographolide sulfonate (AS) is an active component of the traditional herbal medicine *Andrographis paniculata*. This study aims to explore the inhibitory effect and corresponding mechanisms of AS on MRSA and its biofilms. Three doses of AS (6.25, 12.5, and 25 mg/ml) were introduced to MRSA with biofilms. *In vitro* antibacterial testing and morphological observation were used to confirm the inhibitory effect of AS on MRSA with biofilms. Real-time PCR and metabonomics were used to explore the underlying mechanisms of the effect by studying the expression of biofilm-related genes and endogenous metabolites. AS displayed significant anti-MRSA activity, and its minimum inhibitory concentration was 50 μg/ml. Also, AS inhibited biofilms and improved biofilm permeability. The mechanisms are mediated by the inhibition of the expression of genes, such as quorum sensing system regulatory genes (*agrD* and *sarA*), microbial surface components–recognizing adhesion matrix genes (*clfA* and *fnbB*), intercellular adhesion genes (*icaA*, *icaD,* and *PIA*), and a gene related to cellular eDNA release (*cidA*), and the downregulation of five biofilm-related metabolites, including anthranilic acid, *D*-lactic acid, kynurenine, *L*-homocitrulline, and sebacic acid. This study provided valuable evidence for the activity of AS against MRSA and its biofilms and extended the methods to combat MRSA infection.

## Introduction

Methicillin-resistant *Staphylococcus aureus* (MRSA) is one of the most common drug-resistant bacteria, and MRSA infection has posed a serious threat to public health (Fulaz et al., 2020). The formation of MRSA biofilms and their inherent resistance to antibiotics is the root cause of MRSA infection (Zhang et al., 2020). Biofilms are an organized bacterial population that gradually forms during an infection and have the ability to withstand antibiotics (Craft et al., 2019; Cascioferro et al., 2021). A positive relationship has generally been observed between biofilm formation ability and antimicrobial resistance (Sun et al., 2019). Bacteria that attach to a surface and grow as biofilms are protected from antibiotic-induced killing. In addition, reduced antibiotic susceptibility contributes to the persistence of biofilm infections, forming a vicious cycle (Feng et al., 2020). This result explains why MRSA has acquired resistance to practically all antibiotics developed for clinical use in the past 50 years, including vancomycin (Van), which is the last resort to treating MRSA infection (Howden et al., 2010; McGuinness et al., 2017). Researchers have turned their attention to traditional medicine (TM), expecting to identify potential antibacterial drugs that will address the limitation that all antibiotics, including Van, are ineffective at removing mature biofilms (Chopra et al., 2015).

TM mainly originates from natural products. TM exerts its effect through different mechanisms compared to conventional antibiotics, which might be of clinical value in the treatment of infectious diseases associated with biofilms (Ordonez et al., 2011; Borges et al., 2015). *Andrographis paniculata*, a highly abundant natural product and medicinal plant, is regarded as a “natural antibiotic” (Patil and Jain, 2020). Pharmacological studies have shown that it possesses anti-inflammatory, antibacterial, and antipyretic properties (Patil and Jain, 2020) and it has been used to treat a variety of infectious diseases, such as furuncle, upper respiratory tract infection, and chronic pharyngitis caused by MRSA infection. The antibacterial activity is primarily attributed to andrographolide (AG), the active constituent of the herb (Liew et al., 2020). AG exhibits potential antibacterial activity against most Gram-positive bacteria, among which MRSA is the most sensitive to AG (Banerjee et al., 2017). More importantly, AG significantly inhibits biofilm formation without cytotoxicity (Feng et al., 2020). It works by inhibiting virulence factors and the quorum sensing (QS) system, an intercellular communication mechanism closely related to biofilm formation, stability, and development (Solano et al., 2014; Castillo-Juarez et al., 2015). According to previous studies, andrographolide sulfonate (AS), a water-soluble form of silver sulfonate, is the main ingredient in Xiyanping injection. It is more effective in inhibiting bacterial growth and the QS system than the parental compound AG (Zhang et al., 2019a). Xiyanping injection has also long been used in the clinic to treat community-acquired pneumonia, and, compared with azithromycin, it performs better in terms of clinical indicators, such as shorter hospital stays, reduced adverse effects, and time to lung-shadow absorption assessment (Shi et al., 2019). Nevertheless, to date, the specific pharmacodynamic mechanism of AS against MRSA is not known. Therefore, elucidating the therapeutic effect of AS on MRSA infection and the mechanism underlying its efficacy is important for further research on the antibacterial activity of AS.

In the study of drug interventions for MRSA biofilms, choosing the right methodology will provide an important support and ensure the reliability and credibility of the study. Real-time PCR (RT-PCR) is a commonly used method for quantitative analyses of gene expression. In recent years, it has become a standard tool for many laboratories and scientists working in the field of microbial ecology to determine the pathway and characteristics of drug action (Thomas et al., 2012). Metabolomics, an important branch of systematic biology, is mainly used to evaluate the effects of the environment, disease status, or drug intervention on endogenous small-molecule metabolites, such as amino acids, peptides, and lipids (Zhang et al., 2018). It adopts a “top-down” strategy to reflect the function of organisms from terminal symptoms of metabolic networks and understand metabolic changes in a complete system caused by interventions in a holistic context (Wang X. et al., 2011). Metabolomics covers a wide range of substances and focuses on holistic and dynamic evaluation (Zhang et al., 2018), beneficially providing an opportunity to scientifically examine the treatment effect of TM (Wang X. et al., 2011). Recently, metabolomics investigations have been widely used to evaluate the biological efficacy and corresponding mechanism of TM.

In the present study, *in vitro* antibacterial experiments and morphological observations were used to determine the effects of AS on MRSA biofilms. Based on these confirmed effects, RT-PCR was applied to elucidate the expression of biofilm-related genes. Additionally, metabolomics testing was performed to identify novel biomarkers capable of characterizing mature MRSA biofilms and the pharmaceutical mechanism of AS. This study preliminarily elucidated the inhibitory effect and corresponding mechanism of AS on MRSA and MRSA biofilms, which provides valuable evidence and guidance for the clinical diagnosis and therapy of MRSA infection.

## Methods

### Antimicrobial Agents and Chemicals

AS was provided by Jiangxi Qingfeng Pharmaceutical Co., Ltd. (Ganzhou, China). Van hydrochloride injection (lot no. 657) was obtained from EliLilly, Japan. Tryptone and yeast extracts were obtained from Oxoid, United Kingdom, NaCl was obtained from Merck, Germany, and 2,3,5-triphenyltetrazolium chloride (TTC) was obtained from Amresco, United States. 2,3-Bis-(2-methoxy-4-nitro-5-sulfophenyl)-2H-tetrazolium-5-carboxanilide (XTT) and phenazine methosulfate (PMS) were purchased from Sigma-Aldrich, United States.

### Strains and Growth Conditions

MRSA was obtained from samples of MRSA strains that had been identified and isolated in the Laboratory Department of Dongzhimen Hospital in Beijing. MRSA was maintained in cryogenic storage at −80°C on glass beads. Working cultures of bacteria were maintained on the solid LB medium at 4°C and subcultured in the liquid LB medium. In brief, the MRSA strain was cultured with shaking at 280 rpm for 18 h at 37°C and diluted with the LB medium to the starting inoculum with an OD_600_ = 0.02.

### Determination of the Minimum Inhibitory Concentration

The minimum inhibitory concentration (MIC) is defined as the lowest sample concentration that completely inhibits the growth of microorganisms (Fankam et al., 2011). The MIC of AS for MRSA was determined using the microbroth dilution method. The experiment was performed in sterile 96-well plates in a final volume of 200 μl, consisting of 100 μl of microbial cultures (OD_600_ = 0.02) and 100 μl of serially diluted AS (0.1, 0.2, 0.78, 1.56, 3.13, 6.25, 12.5, 25, and 50 mg/ml). Van (0.25, 0.5, 1, 2, 4, 8, 16, 32, 64, and 128 μg/ml) was used as a positive control drug. Microplates were incubated at 37°C for 24 h, followed by the addition of 20 μl of 2.5 mg/ml TTC and incubation for an additional 20 min at 37°C. Viable bacteria reduced the yellow dye to pink.

### Biofilm Assays

The method for biofilm quantification was performed as described previously and modified as described by Wang Y. et al., 2011. We used the LB medium containing 0.25% glucose (LB-G) to dilute the MRSA bacterial solution to an OD_600_ = 0.1. The diluted culture was transferred to a sterile 96-well plate and incubated at 37°C and 80% RH for 24 h. One hundred microliters of AS (6.25, 12.5, and 25 mg/ml) was added, and the well containing the medium was used as a negative control well. After incubating the cultures at 37°C and 80% RH for 24 h, the medium and planktonic cells were removed and the wells containing the biofilm were washed with 0.9% NaCl. Then, the XTT reduction assay was performed to evaluate the viability of the biofilms. For this experiment, 40 μl of the XTT-PMS mixed solution (XTT:PMS = 1:100) was added to each well. The 96-well plate was incubated in the dark at 37°C for 20 min, and then, the optical density was measured at 490 nm using a microplate reader. A decrease in the number of live cells correlates with a decrease in the overall activity of the dehydrogenases responsible for transforming the sodium salt of XTT into formazan, which was determined colorimetrically (Yang et al., 2018).

### Confocal Laser Scanning Microscopy

MRSA (OD_600_ = 0.1) diluted with LB-G was transferred to a sterile Petri dish and incubated at 37°C and 80% RH for 24 h. After the planktonic bacteria were removed, 1 ml of 25 mg/ml AS and 4 μg/ml Van were added and LB-G was used as a negative control. After incubating at 37°C and 80% RH for 24 h, the supernatant was removed. Three fluorescent dyes were used for staining. SYTO-9 and PI from the LIVE/DEAD®BacLight™ Bacterial Viability Kit (cat. no. L7012) and Matrix-staining solution were used to measure the extracellular matrix of biofilms. The ratio of the three dye solutions was 1.5:1.5:1,000. The biofilm was stained in the dark for 20 min. Then, the dye was removed, and 500 μl of LB-G was added to each well. The tomographic images, three-dimensional reconstruction images, and images of the biofilm thickness were captured using an Olympus TM FluoView FV1000 microscope to determine the spatial distribution of living/dead bacteria in the biofilm structure. The samples were placed under a confocal laser scanning microscope and observed with a ×10 objective at the different excitation wavelengths of SYTO-9 (488 nm), PI (559 nm), and Matrix (559 nm) stains. SYTO-9 quickly penetrates the cell wall and binds to DNA with green fluorescence; therefore, it characterizes living bacteria. On the other hand, PI does not penetrate the intact cell wall but binds to the mixture of damaged cell DNA and extracellular DNA with red fluorescence to characterize the dead bacteria.

### Scanning Electron Microscopy

The biofilm samples were prepared on glass cover slides using the method described above. The sample was fixed with 4% glutaraldehyde for 24 h at 4°C and then fixed with 1% osmic acid for 1 h. After gradual dehydration with ethyl alcohol solutions (60, 70, 80, 90, 95, and 100%), the sample was freeze-dried. The specimens were then sputter-coated with gold. Finally, the cells were observed under a scanning electron microscope.

### Detection of Biofilm Permeability

The change in biofilm permeability was judged by observing the change in the size of the bacteriostatic zone caused by the filter paper with the increase in the time required to absorb Van from agar plates covered by biofilms. The change in the permeability of MRSA biofilms treated with AS and Van was detected by establishing a filter paper–biofilm–antibiotic agar plate model (Anderl et al., 2000). MRSA biofilms were cultured on round aseptic paper in the LB-G medium and incubated with 12.5 mg/ml AS, 25 mg/ml AS, or 4 μg/ml Van for 24 h. At the same time, the LB-G medium was used as a negative control group. The treated biofilm was transferred to an LB agar plate containing 500 μg/ml Van, and a round 6 mm wet filter paper was placed on the biofilm. If the biofilm structure is destroyed, the Van from the agar plate will be adsorbed on the filter paper according to the permeation principle. After incubating at 37°C and 80% RH for 2, 4, and 6 h, the round filter paper was transferred to an LB agar plate covered with MRSA. The size of the bacteriostatic zone was measured after incubating at 37°C and 80% RH for 18 h.

### Detection of the Expression of MRSA Biofilm–Related Genes Using Real-Time PCR

#### Extraction of Total RNA

The relative quantitative analysis of gene expression in planktonic MRSA and mature MRSA biofilms was carried out using RT-PCR. MRSA cells were cultured with LB-G at 37°C and 280 rpm for 0 and 24 h, respectively, and then incubated with 25 mg/ml AS at 37°C and 80% RH for 24 h. The cells were then harvested by centrifugation and immediately suspended in RNAlater (Ambion, Austin, TX). RNA was then purified with the SV Total RNA Isolation system (Promega, Madison, WI), followed by DNaseI treatment (Turbo DNA-free kit; Ambion) to eliminate residual contamination of genomic DNA. The purity and concentration of the RNA were determined by spectrophotometry and gel electrophoresis. Reverse transcription reactions were performed with the High-Capacity Complementary DNA (cDNA) Reverse Transcription Kit (ABI, Foster City, CA).

#### Real-Time PCR

PCR primers (Table 1) were designed with Primer Express Software Version 3.0 (ABI). Amplification of cDNA (1 ng) was performed with 250 nM gene-specific primers and a PowerSYBR Green Master Mix (ABI). PCR amplification, detection, and analyses were performed using the StepOne RT-PCR system (ABI). The critical threshold cycle was defined for each sample, and expression levels were normalized to the 16S rRNA gene as an internal standard; 16S rRNA levels did not vary under our experimental conditions. Each assay was performed in triplicate.

**TABLE 1:** Primer sequences used for the quantitative analysis.

Primer name	Primer sequence (5’→3′)	Accession number
*agrD*-F	TCA​TTT​TTT​GAT​TTT​ATA​ACT​GGT​G	AF288215.1
*agrD*-R	TCT​TTA​GGT​ATT​TCA​ACT​TCG​TCC	
*cidA*-F	TAA​CTT​GGG​TAG​AAG​ACG​GTG​C	NC_022604
*cidA*-R	CGT​CTA​CAC​CTT​TAC​GAT​GTT​TAT​G	
*clfA*-F	ATT​GGC​GTG​GCT​TCA​GTG​CT	CP076105.1
*clfA*-R	CGT​TTC​TTC​CGT​AGT​TGC​ATT​TG	
*fnbB*-F	AGG​AAG​AAG​CGA​AAC​CTC​AAG	CP076105
*fnbB*-R	ATG​CCC​CTC​AAT​AGA​ACC​AAT	
*icaA*-F	CGA​AGT​CAG​ACA​CTT​GCT​GG	CP076105.1
*icaA*-R	GCT​TCC​AAA​GAC​CTC​CCA​A	
*icaD*-F	GGT​CAA​GCC​CAG​ACA​GAG​G	CP076027.1
*icaD*-R	ACA​CAC​GAT​ATA​GCG​ATA​AGT​GC	
*PIA*-F	CCT​ATC​CTT​ATG​GCT​TGA​TGA​AT	NC_007795.1
*PIA*-R	TAA​TAA​TCA​TTG​GAG​TTC​GGA​GTG	
*sarA*-F	ATG​GGG​AAC​ATG​ATC​CTT​TG	CP076105.1
*sarA*-R	TAG​CCG​CAT​AAC​GAG​CAG​TA	
16S rRNA-F	TTC​TGG​TCT​GTA​ACT​GAC​GCT​G	CP071594.1
16S rRNA-R	CGA​AGG​GGA​AGG​CTC​TAT​CT	

### UPLC-TOF-MS Detection of Metabolites in MRSA Biofilms

#### Metabonomics Sample Preparation

Nontargeted metabonomics was conducted, and the mechanism underlying the effect of 25 mg/ml AS on MRSA was discussed. Twenty-five millilitres of a MRSA solution with an OD_600_ = 0.1 was added to two sterile 250 ml conical flasks. An overnight biofilm culture was prepared by incubating the sample in a constant-temperature concussion box at 37°C and 180 rpm for 24 h. The AS (25 mg/ml) and blank LB medium (50 ml) were added to the conical flask on the next day. Fifty milliliters of the MRSA cultures at an OD_600_ = 0.1 and the same volume of blank LB medium were added to another conical flask. After 24 h of coincubation, the same volume of 60% methanol in water was added at −20°C for cold methanol extraction. The sample was centrifuged at 3,000 rpm for 10 min at 4°C. Then, the supernatant was removed, and the precipitate was stored at −80°C until metabolite extraction.

#### Extraction and Analysis of Metabolites

After thawing at room temperature, 300 μl of methanol was added to the sample. Proteins were precipitated with methanol, fully swirled for 30 s, and centrifuged at 12,000 rpm for 15 min at 4°C. An equal volume of each sample was combined and used as a quality control (QC) sample.

The instrument analysis platform was LC-Q/TOF-MS (Agilent, 1290 Infinity LC, 6545 UHD, and Accurate-Mass Q-TOF/MS), and the separation column was a C18 column (Agilent, 100 mm × 2.1 mm, 1.8 μm). The chromatographic separation conditions are described as follows. The column temperature was 40°C. The flow rate was 0.4 ml/min. The mobile phase consisted of A, water+0.1% formic acid, and B, acetonitrile+0.1% formic acid. The injection volume was 4 μl, and the temperature of the automatic injector was 4°C. The LC/MS data were preprocessed using Mass Profiler software (Agilent) and edited later in EXCEL2007 software. The final results were organized into a two-dimensional data matrix, including variables (retention time and mass-to-charge ratio), observation quantity (sample), and peak intensity.

The edited data matrix was imported into SIMCA-P software (Umetrics AB, Umea, Sweden, version 13.0) for principal component analysis (PCA). Statistical analyses were performed using the free online tool MetaboAnalyst 3.0. In brief, the exported table of putative metabolites with a median RSD of <0.2 (20%) within the QC group and a confidence level of >5 was uploaded to MetaboAnalyst 3.0. Data with >50% missing values were removed, and the remaining missing values were replaced with a small value (half the minimum positive value in the original data) (Wehrens and Salek, 2016). Data were filtered using the interquartile range, normalized to the median, log2 transformed, and autoscaled (mean centered and divided by the standard deviation of each variable). One-way ANOVA and Fisher’s least square difference (LSD) test were used to identify the significantly perturbed metabolites between the 25 mg/ml AS-treated and control groups. Statistically and significantly altered metabolites were selected using a false discovery rate of <0.05 for one-way ANOVA and a *p* value of <0.05 for Fisher’s LSD test.

## Results

### Antibacterial Activity of Andrographolide Sulfonate and Vancomycin Against MRSA

**FIGURE 1:**
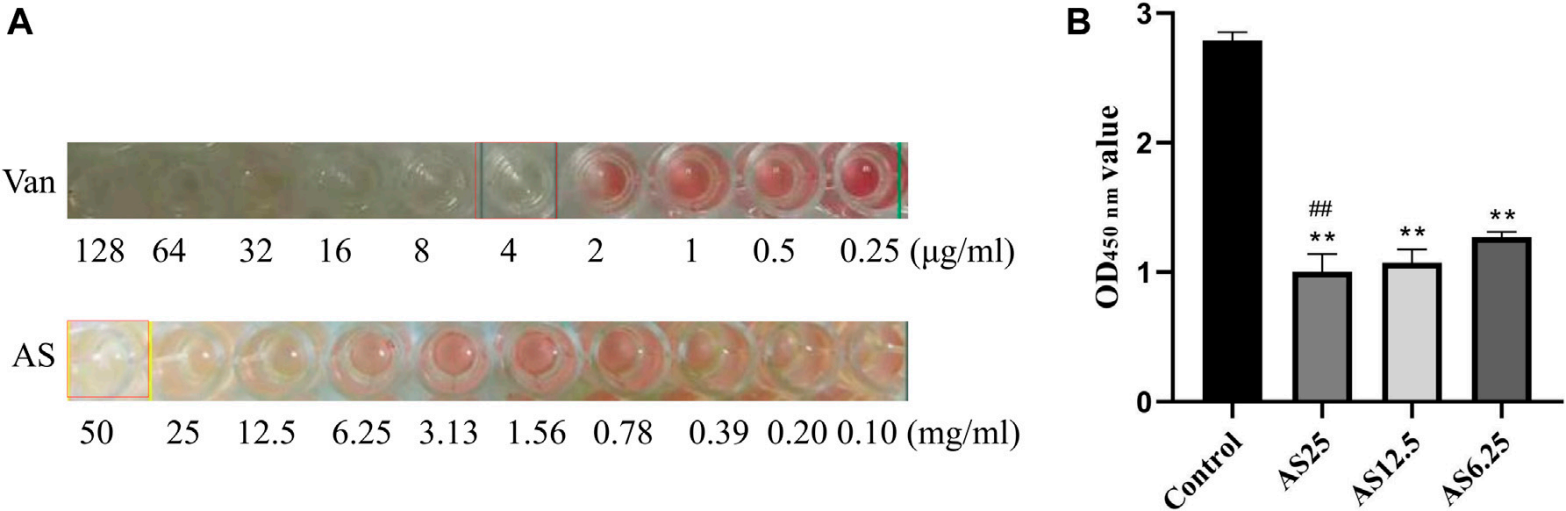
Detection of the MIC of each drug for MRSA biofilms. **(A)** The MICs of Van and AS for MRSA. **(B)** Effects of different concentrations of AS on living bacteria in MRSA biofilms (**p < 0.01, compared with the control).

MRSA strains have different sensitivity rates to AS and Van. As shown in Figure 1A, Van exerted a bacteriostatic effect on MRSA at 4 μg/ml, AS had no bactericidal activity in the dose range of 0.1–25 mg/ml, and 50 mg/ml AS exerted the same inhibitory effect on MRSA as 4 μg/ml Van. The MIC of AS for MRSA is 50 mg/ml.

### Andrographolide Sulfonate Inhibits MRSA Biofilm Formation

Based on the MIC results of AS, three concentrations of AS, 1/8 MIC, 1/4 MIC, and 1/2 MIC (6.25, 12.5, and 25 mg/ml), were selected for the XTT reduction assay on MRSA biofilms. Compared with the untreated control biofilm, the effect on inducing biofilm cell death was stronger as the dose of AS increased (Figure 1B). The concentration of AS that produced the best inhibitory effect was 25 mg/ml. This concentration was used as the dose in our subsequent experiments.

**FIGURE 2:**
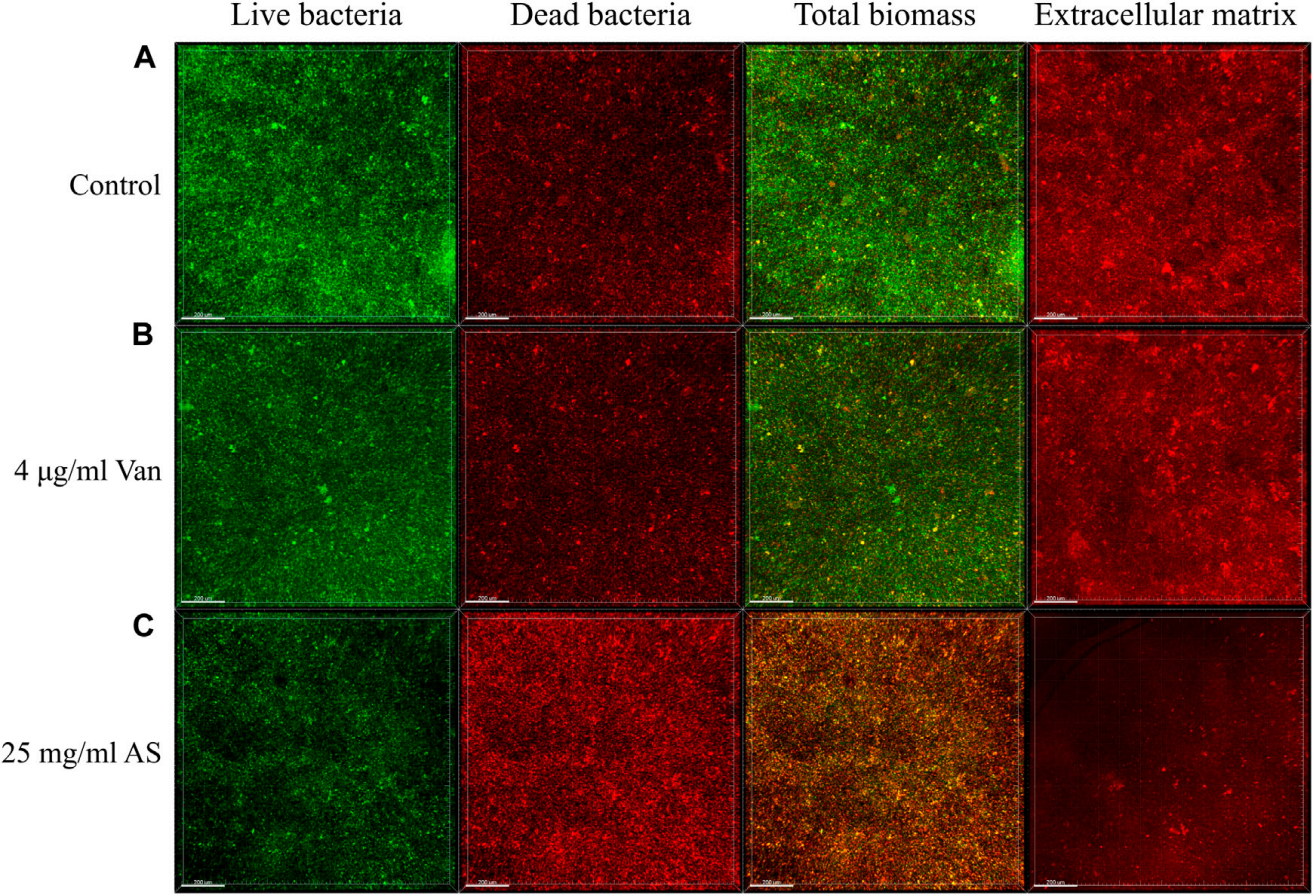
CLSM micrographs of MRSA biofilms treated with AS and Van. In the first three columns, green represents the fluorescence signal of living bacteria and red represents the fluorescence signal of dead bacteria. The total biomass is the superposition of the two fluorescence signals, which indicates the corresponding biomass distribution of living bacteria and dead bacteria. The fourth column shows the distribution of the extracellular matrix fluorescence signal. The scale bar in images represents 200 μm.

Confocal laser scanning microscopy (CLSM) observations and digital image analysis showed that the surface of the control group was rough, the fluorescence signal of living bacteria was strong, and the whole Petri dish was covered with many vigorous microbial colonies (Figure 2). The condition of the 4 μg/ml Van-treated group was similar to that of the control group. The fluorescence signal of living bacteria in the culture containing 25 mg/ml AS was weaker, and the number of colonies was substantially reduced. In addition, the fluorescence signal of dead bacteria was much higher than that of the former two groups. By staining the extracellular matrix with a Matrix-staining kit, the extracellular matrix content of the MRSA biofilm treated with 25 mg/ml AS was significantly lower than that of the 4 μg/ml Van-treated group. AS reduced the extracellular matrix content and inhibited the growth of living bacteria in the biofilm.

**FIGURE 3:**
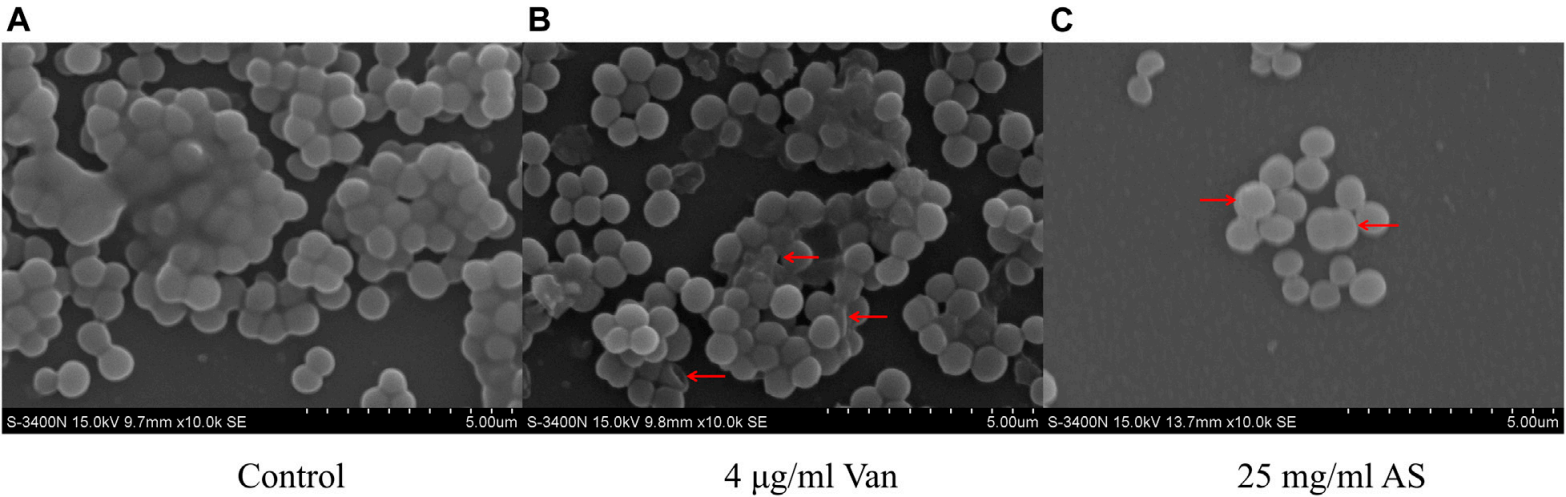
SEM micrographs of MRSA biofilms treated with AS and Van. **(A)** Normal MRSA biofilms. **(B)** Abnormally shaped cells indicated by red arrows. **(C)** Cells with abnormal division indicated by a red arrow.

Scanning electron microscopy (SEM) observations at ×10000 magnification revealed that the biofilm structure of the control group was dense and massive colonies were growing on glass cover slips (Figure 3). The size of the cells was uniform, the shape was normal, and the surface was smooth. When MRSA was treated with 4 μg/ml Van, the structure of the biofilm was not as dense as that of the control group. Individual bacteria were damaged and presented an empty shell state after the outflow of the intracellular material. The structure of the biofilm almost completely disappeared in the electron microscopy image of the 25 mg/ml AS-treated group. Abnormal dichotomy and an increase in the volume were observed in the bacteria.

### Effects of Vancomycin and Andrographolide Sulfonate on the Permeability of MRSA Biofilms

**FIGURE 4:**
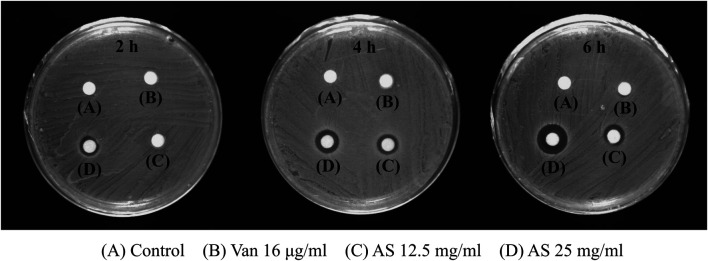
Effects of AS and Van on the permeability of MRSA biofilms. The 16 μg/ml Van-treated group was the same as control group at all three time points and there was no inhibition zone. The 12.5 mg/ml AS showed inhibition zone at 4 h and 6 h, which was weak. The inhibition zone increased with an increasing duration of action of 25 mg/ml AS (6 h > 4 h > 2 h).

In the control group, no inhibition zone was observed at 2, 4, and 6 h, indicating that the biofilm permeability was low without drug intervention. No inhibition zone was detected in the 16 μg/ml Van-treated group, which indicates that Van did not change the permeability of MRSA biofilms. However, 12.5 mg/ml AS produced an inhibition zone after 4 h and 25 mg/ml AS produced an inhibition zone after 2 h. Thus, AS improved the permeability of MRSA biofilms, and this effect occurred faster with increase in concentrations (Figure 4).

### Effect of Andrographolide Sulfonate on MRSA Gene Expression

**FIGURE 5:**
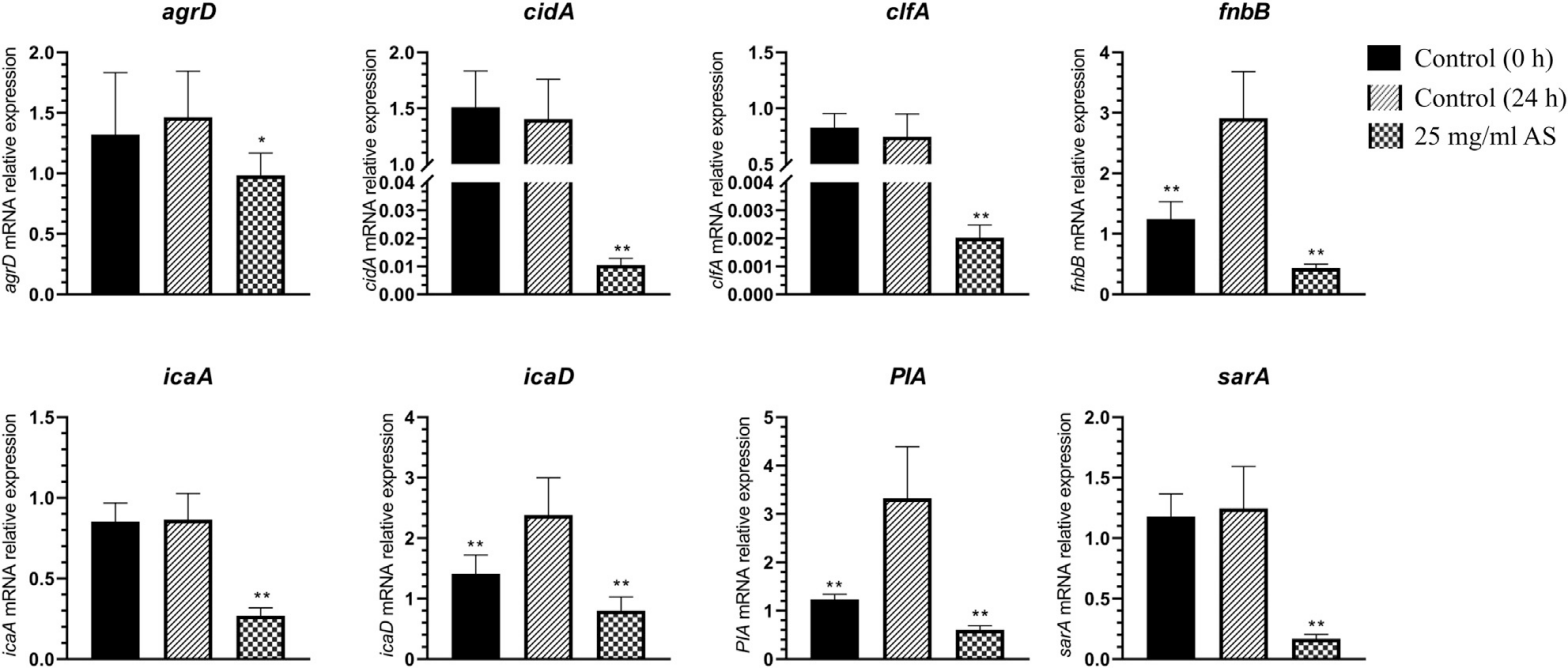
Changes in MRSA gene expression. *p < 0.05, compared with the control (24 h); **p < 0.01, compared with the control (24 h).

The expression of the adhesion-related genes, intercellular adhesion (*ica*) *D*, fibronectin binding (*fnb*) *B,* and polysaccharide intercellular adhesion (*PIA*), was significantly increased in MRSA biofilms, suggesting that these genes are closely associated with MRSA biofilm formation (Figure 5). When the mature MRSA biofilm was treated with 25 mg/ml AS, the expression of these genes was significantly inhibited compared with that of the control (24 h) (Figure 5).

The expression of staphylococcal accessory regulator (*sar*) *A* (*p* < 0.01) and accessory gene regulator (*agr*) *D* (*p* < 0.05) related to the QS system was downregulated to different degrees. The expression of *icaA*, clumping factor (*clf*) *A,* and *cidA* was also significantly downregulated after treatment with 25 mg/ml AS (*p* < 0.01).

### Principal Component Analysis

**FIGURE 6:**
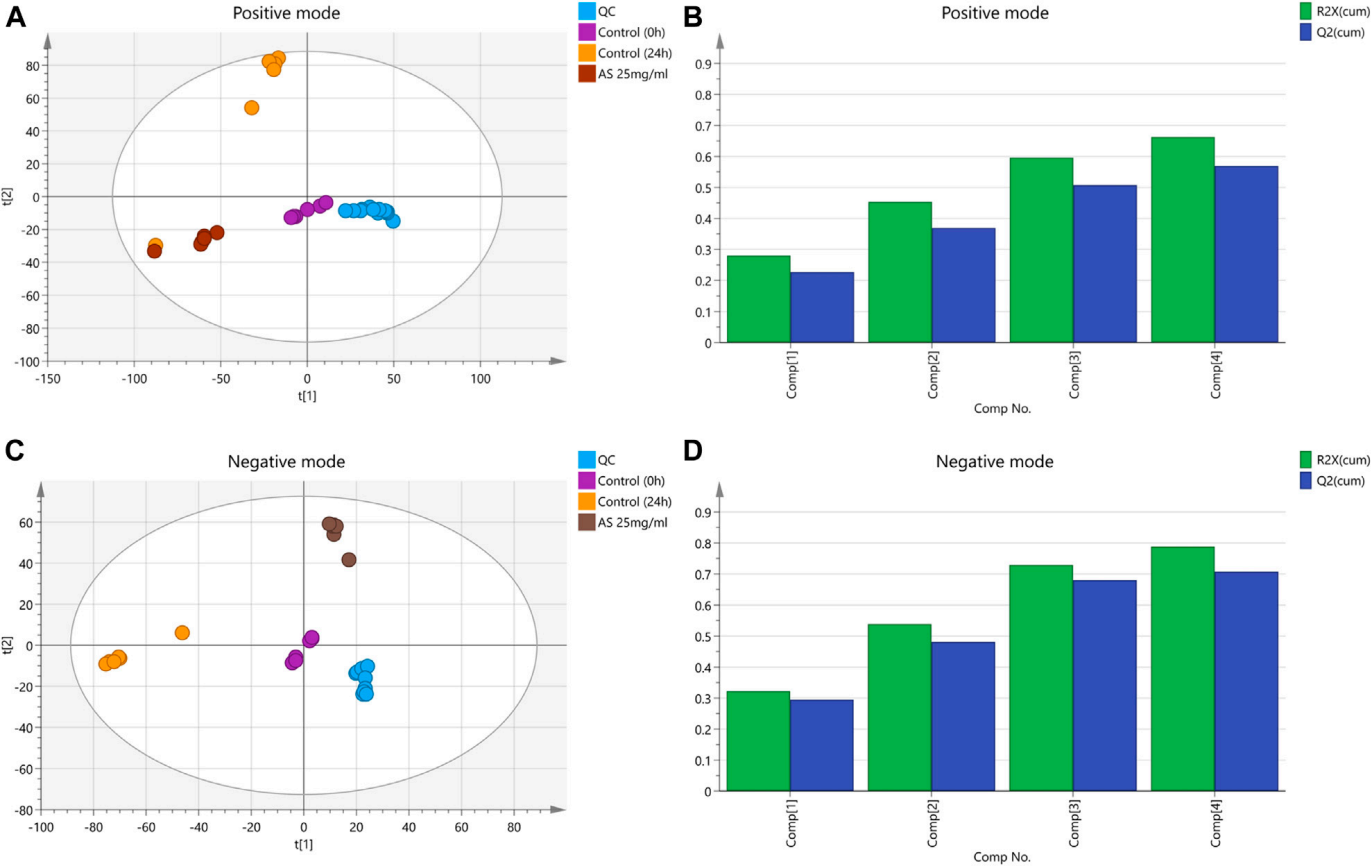
PCA score plots based on the MRSA endogenous metabolic spectra obtained in the positive and negative ion mode. **(A** and **C)** PCA score plots; QC: quality control. **(B)** R2X = 0.663 cum, Q2 = 0.569 cum. **(D)** R2X = 0.788 cum, Q2 = 0.707 cum.

In this analysis, 5 principal components were obtained in the positive mode, with R2X = 0.663 and Q2 = 0.569. In the negative mode, 8 principal components were obtained, with R2X = 0.788 and Q2 = 0.707. The PCA score chart is shown in Figure 6. Samples from the control group at 0 and 24 h were divided into two groups with significant separation trends, indicating that the metabolite profiles of planktonic MRSA and MRSA biofilms were significantly different. The metabolite profiles of the AS-treated group were significantly separated from those of the 24 h control group, indicating a significant effect of AS on perturbing the metabolism of MRSA biofilms.

### Changes in Endogenous-Terminal Small-Molecule Metabolites Before and After the Formation of MRSA Biofilms

**FIGURE 7:**
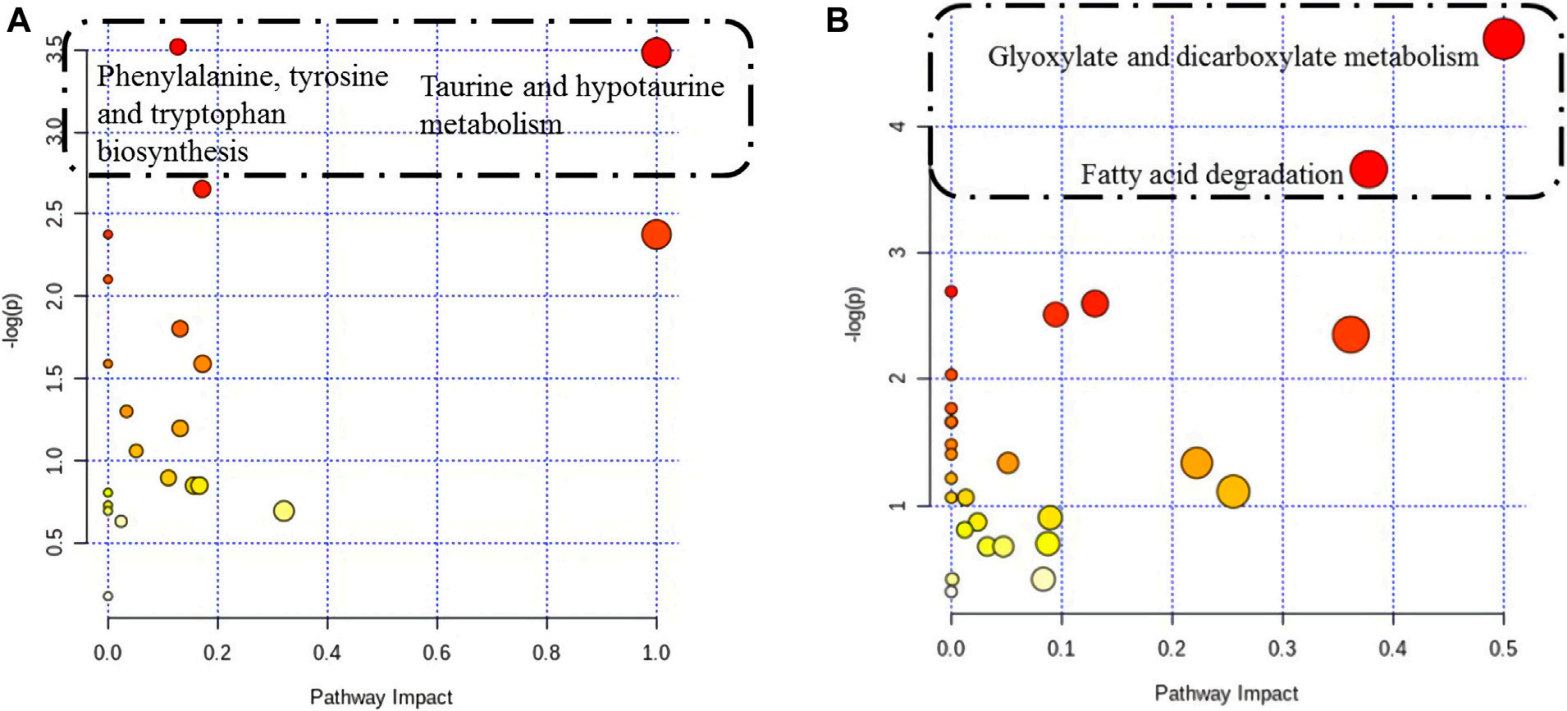
Pathway analysis of differentially altered metabolites in MRSA. **(A)** Overview of the pathway analysis of the 27 biofilm biomarkers. **(B)** Overview of the pathway analysis of the pharmaceutical markers of MRSA biofilms treated with 25 mg/ml AS. The pathway analysis of metabolites with significant differences was performed using MetaboAnalyst 3.0. The bubble chart of the pathway enrichment analysis shows all matched pathways according to the pathway impact values (*X*-axis) of the topological analysis and *p*-values (*Y*-axis) of the pathway enrichment analysis. Each node represents a biological pathway. The bubble area (radius) is proportional to the impact value of each pathway, with the color denoting the significance from the highest in red to the lowest in white. The nodes in the dashed box represent the biological pathways that are significantly related to biofilm markers.

Twenty-seven differentially altered metabolites were identified in the two control groups before and after biofilm formation (Table 2). These metabolites reflect the metabolic phenotypic characteristics of MRSA biofilm formation. The metabolic pathway analysis showed that phenylalanine, tyrosine, and tryptophan biosynthesis and taurine and hypotaurine metabolism were related to these metabolites (*p* < 0.05) (Figure 7A).

**TABLE 2:** Characterization of terminal metabolites in MRSA biofilms.

*n*	RT (min)	Exact mass	Formula	Compound	KEGG	Fold change
1	5.96	210.0753	C_8_H_10_N_4_O_3_	1,3,7-Trimethyluric acid	C16361	5.925
2	1.1	891.204	C_29_H_48_N_7_O_17_P_3_S	2E-Octenoyl-CoA	C05276	−6.914
3	5.36	851.1727	C_26_H_44_N_7_O_17_P_3_S	2-Methylbutyryl-CoA	C01033	7.369
4	1.09	971.1575	C_32_H_44_N_7_O_20_P_3_S	2-Succinylbenzoyl-CoA	C03160	−7.372
5	4.74	160.0974	C_7_H_13_NO_3_	3-Dehydrocarnitine	C02636	−9.932
6	0.9	139.9875	C_2_H_5_O_5_P	Acetyl phosphate	C00227	10.32
7	0.67	559.0717	C_15_H_23_N_5_O_14_P_2_	ADP-ribose	C00301	−5.387
8	0.79	137.0477	C_7_H_7_NO_2_	Anthranilic acid	C00108	6.859
9	1.75	329.0525	C_10_H_12_N_5_O_6_P	cAMP	C00575	4.493
10	0.84	111.0433	C_4_H_5_N_3_O	Cytosine	C00380	−6.288
11	0.73	103.0633	C_4_H_9_NO_2_	D-2-Aminobutyric acid	C02261	−4.792
12	0.71	307.0569	C_9_H_14_N_3_O_7_P	dCMP	C00239	6.882
13	0.72	90.0317	C_3_H_6_O_3_	*d*-Lactic acid	C00256	4.197
14	0.73	147.0532	C_5_H_9_NO_4_	Glutamate	C00025	−7.229
15	0.76	111.0796	C_5_H_9_N_3_	Histamine	C00388	−3.219
16	4.84	208.0848	C_10_H_12_N_2_O_3_	Kynurenine	C01718	3.41
17	0.76	180.0634	C_7_H_14_O_5_	*L*-(-)Sorbose	C00247	3.127
18	0.77	161.1052	C_7_H_15_NO_3_	*L*-Carnitine	C00318	−4.065
19	6.99	189.1113	C_7_H_15_N_3_O_3_	*L*-Homocitrulline	C02427	3.616
20	1.13	181.0739	C_9_H_11_NO_3_	*L*-Tyrosine	C00082	5.083
21	4.7	594.3417	C_33_H_46_N_4_O_6_	*L*-Urobilin	C05793	3.156
22	5.54	207.0895	C_11_H_13_NO_3_	N-Acetyl-d-phenylalanine	C05620	4.615
23	1.14	164.0473	C_9_H_8_O_3_	Phenylpyruvic acid	C00166	7.009
24	0.85	129.0426	C_5_H_7_NO_3_	Pyroglutamic acid	C01879	−7.133
25	0.9	202.1205	C_10_H_18_O_4_	Sebacic acid	C08277	3.932
26	1.07	342.1162	C_12_H_22_O_11_	Sucrose	C00089	6.765
27	0.75	205.0375	C_10_H_7_NO_4_	Xanthurenic acid	C02470	11.37

### Effect of Andrographolide Sulfonate on Endogenous-Terminal Small-Molecule Metabolites in MRSA Biofilms

When biofilms matured, twenty differentially altered metabolites were detected in the MRSA strain from the 25 mg/ml AS-treated group compared with those of the control group (Table 3). These metabolites reflected the pharmacodynamic characteristics of 25 mg/ml AS-treated MRSA biofilms and were related to the two most significant pathways, glyoxylate and dicarboxylate metabolism and fatty acid degradation (Figure 7B).

**TABLE 3:** Characteristics of terminal metabolites in MRSA biofilms treated with 25 mg/ml AS.

*n*	R.T (min)	Exact mass	Formula	Compound	KEGG	Fold change
1	1.03	206.0427	C_7_H_10_O_7_	2-Methylcitric acid	C02225	−3.792
2	N/A	120.0245	C_4_H_8_O_2_S	3-(Methylthio)propionic acid	C08276	−6.704
3	N/A	853.152	C_25_H_42_N_7_O_18_P_3_S	3-Hydroxy-2-methylpropanoyl-coenzyme A	C04047	−9.042
4	3.71	809.1258	C_23_H_38_N_7_O_17_P_3_S	Acetyl-coenzyme A	C00024	10.427
5	0.79	137.0477	C_7_H_7_NO_2_	Anthranilic acid	C00108	−4.071
6	0.84	192.027	C_6_H_8_O_7_	Citric acid	C00158	−3.894
7	4.19	921.251	C_31_H_54_N_7_O_17_P_3_S	Decanoyl-coenzyme A	C05274	−5.859
8	8.19	301.2981	C_18_H_39_NO_2_	*D*-Erythro-dihydrosphingosine	C00836	7.378
9	0.72	90.0317	C_3_H_6_O_3_	*D*-Lactic acid	C00256	−3.451
10	5.1	588.2948	C_33_H_40_N_4_O_6_	*D*-Urobilin	C05795	−6.841
11	4	1,061.407	C_41_H_74_N_7_O_17_P_3_S	Eicosanoyl-coenzyme A	C02041	5.026
12	5.54	207.0895	C_11_H_13_NO_3_	Homocitrulline	C02427	−4.92
13	0.28	268.0808	C_10_H_12_N_4_O_5_	Inosine	C00294	−8.471
14	6.05	189.0426	C_10_H_7_NO_3_	Kynurenic acid	C01717	−13.006
15	5.41	208.0848	C_10_H_12_N_2_O_3_	Kynurenine	C01718	−7.402
16	1.6	105.0426	C_3_H_7_NO_3_	*L*-Serine	C00065	−3.434
17	4.45	977.3136	C_35_H_62_N_7_O_17_P_3_S	Myristoyl-coenzyme A	C02593	−12.865
18	0.9	202.1205	C_10_H_18_O_4_	Sebacic acid	C08277	−13.467
19	1.95	379.2488	C_18_H_38_NO_5_P	Sphingosine-1-phosphate	C06124	−9.565
20	1.07	342.1162	C_12_H_22_O_11_	Sucrose	C00089	−7.884

Five common terminal metabolites, anthranilic acid, *D*-lactic acid (*D*-LA), kynurenine (KYN), *L*-homocitrulline, and sebacic acid, were identified in the control group and AS group (Table 4). Their levels increased during biofilm formation but decreased significantly after treatment with 25 mg/ml AS.

**TABLE 4:** Common terminal metabolites reflecting biofilm formation and AS efficacy.

*n*	Compound	KEGG	Fold change	
			Control	25 mg/ml AS
1	Anthranilic acid	C00108	6.859	−4.071
2	*D*-Lactic acid	C00256	4.197	−3.451
3	Kynurenine	C01718	3.41	−7.402
4	*L*-Homocitrulline	C02427	3.616	−4.92
5	Sebacic acid	C08277	3.932	−13.467

## Discussion

The treatment of MRSA infection has become a clinically difficult problem. Biofilm formation plays a key role in MRSA infection because the formation of the biofilm provides increased protection of bacteria from antibiotics and host defenses (Chao et al., 2017). Therefore, interfering with the formation of biofilms would be a sensible approach when developing a new treatment for recalcitrant MRSA infections. Currently, the extracts of traditional medicine have become a research hotspot in the development of new antimicrobials. The antibacterial activity of andrographolide, the active component of AG, has been widely studied (Zhang et al., 2019b). However, the absolute bioavailability of AG was reported to be only 2.67% according to a previous study, which substantially limits its clinical efficacy due to its poor water solubility and susceptibility to extracellular excretion by P-glycoprotein (Ye et al., 2011). By transforming hydroxyl groups into sulfate, the sulfation reaction is an effective method to obtain soluble derivatives with both improved water solubility and bioavailability. The main ingredient of Xiyanping injection is AS, which is modified by AG sulfonation. Several pharmacokinetic studies have shown that AS has a long *t*_1/2_ (drug half-life), shows considerable metabolic stability, and is rapidly absorbed and distributed to the kidney, colon, liver, and lung tissues (Chong et al., 2013; Lan et al., 2013; Zheng et al., 2016). Safety studies of AS are of equal clinical interest. Only a few randomized occurrences of immediate hypersensitivity reactions to Xiyanping have been reported. A 2017 clinical report cited a 0.12% incidence of adverse reactions following Xiyanping injection (Zhao et al., 2017). Computer analyses of ligand–protein interactions also indicate a very low risk of potential allergic reactions to AS (Yang et al., 2019). The excellent efficacy performance of AS in the treatment of biofilm-mediated infections is under close scrutiny in current anti-infective research (Jiang et al., 2009; Simoes et al., 2009; Ordonez et al., 2011). Nevertheless, the mechanisms underlying the inhibitory effect of AS on MRSA biofilms are still not completely understood. Hence, the therapeutic effect of AS and the precise underlying mechanisms must be elucidated to facilitate the clinical application of AS in the treatment of MRSA infection. We are the first group to document the inhibitory effect of AS on MRSA biofilms and discover the terminal metabolites of the corresponding mechanism through metabolomics. In our experiments, biofilms of MRSA strains isolated from the clinic and MRSA broth supplemented with AS and Van, respectively, were formed *in vitro* after a 24 h incubation of bacteria in the LB medium at room temperature. The expression of genes associated with virulence factors and biofilms was measured using RT-PCR to reveal the association between changes in gene expression and MRSA biofilm formation and AS efficacy. The pharmaceutical mechanisms of AS were identified by performing metabolomics profiling of the corresponding metabolites. Due to the characteristics of TM working mechanisms, metabolomics is particularly suitable for pharmacodynamic studies. Small-molecule metabolites play an important role in biological systems and represent attractive candidates to understand the phenotypes of drug effects. In particular, highly sensitive and specific terminal biomarkers in MRSA biofilms are very useful for a comprehensive study of the efficacy of TM. Therefore, in the present study, a metabolomics approach was employed to determine the subtle change in the metabolite profile of the AS intervention group, explore pharmaceutical biomarkers, and reveal the underlying mechanisms. Meanwhile, the metabolic networks and pathways involved were subjected to KEGG analyses.

*In vitro* experiments showed a substantial difference in the effective MIC of AS for MRSA compared with Van. Compared with the traditional antibiotic, Van, a higher dose of AS is required to kill planktonic MRSA. However, MRSA can exist in both the planktonic form and biofilms, with the latter being predominant in clinical practice (McCarthy et al., 2015). The formation of biofilms contributes to the ineffective eradication of MRSA infection. When bacteria produce dense biofilms, the situation changes considerably. Matrix components such as PIA and extracellular DNA (eDNA) on the biofilms are important for the bacteria to evade the host immune response and antibiotic attack (Harapanahalli et al., 2015). In the inhibition experiment of AS against MRSA biofilms, only 1/2 MIC of AS inhibited MRSA biofilms. Overall, AS showed a greater advantage in inhibiting MRSA biofilms than planktonic MRSA. In addition, the inhibitory effect was concentration dependent. This discovery extends the general understanding of the antibacterial activity of TM. Our hypothesis that when MRSA biofilms are exposed to the MIC of Van and 1/2 MIC of AS, the 1/2 MIC of AS will cause more significant changes in the physical properties and function of MRSA biofilms and inhibit their growth was confirmed both in biofilm permeation experiments and morphological observations. In addition, SEM showed that AS not only reduced the content of MRSA biofilms but also interfered with the normal cell division of MRSA. This compound inhibited the proliferation of MRSA to a certain extent. However, the specific mechanism requires further studies.

The MRSA biofilm is a microbial colony with a complete structure. The formation of biofilms involves four stages, adhesion, aggregation, maturation, and diffusion, and the process is regulated by virulence factors, regulatory factors, and the QS system. We detected the expression of related genes in planktonic MRSA and mature MRSA biofilms. Notably, sets of genes were upregulated during biofilm formation, namely, *fnbB*, *icaD,* and *PIA*, which mainly contained some known key genes related to adhesion. These key genes may distinguish the biofilm from planktonic MRSA.

**FIGURE 8:**
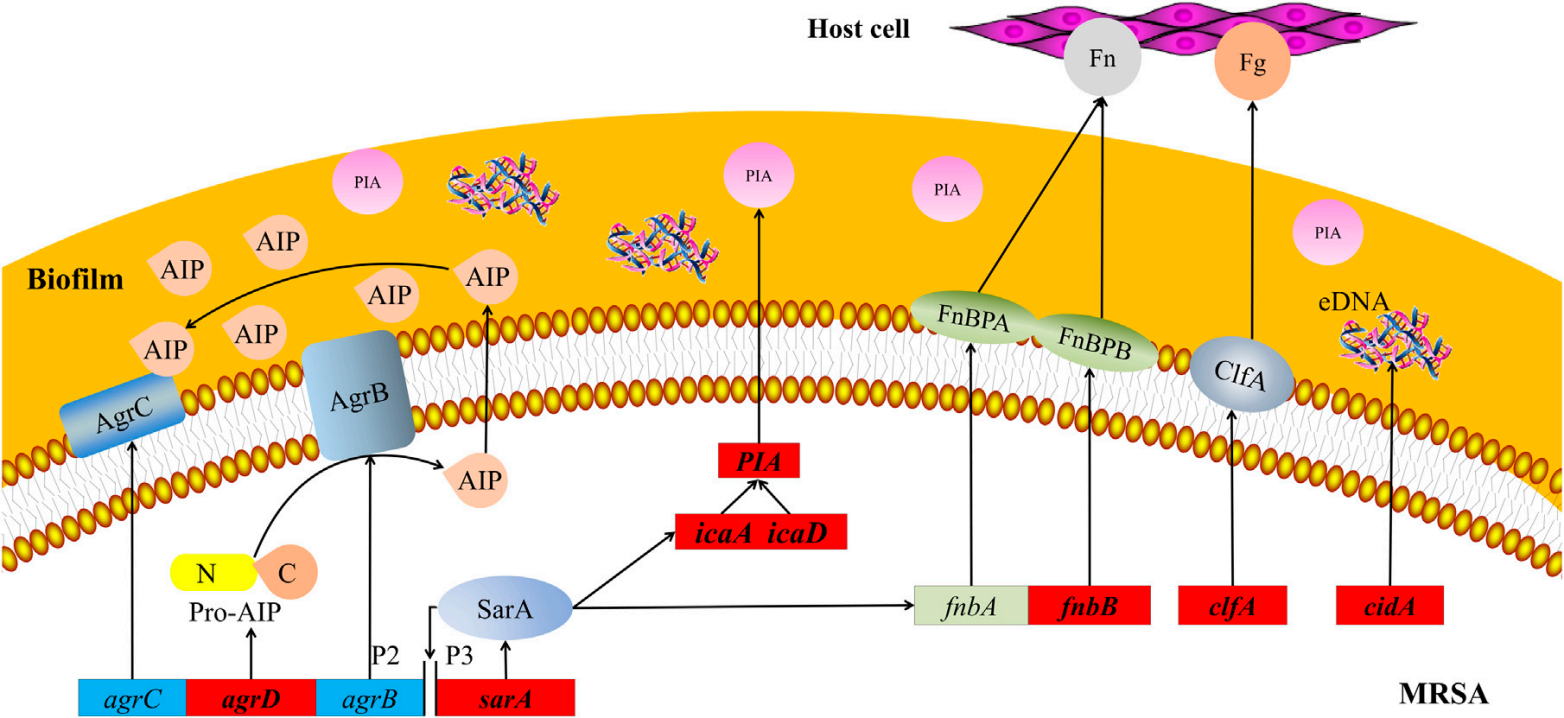
Schematic map of pathways related to MRSA biofilms. Red boxes represent MRSA genes that are downregulated by treatment with 25 mg/ml AS. *agrB*, *agrC,* and *agrD*, accessory gene regulators B, C, and D; AIP, autoinducer peptide; *clfA* and *clfB*, clumping factors A and B; eDNA, extracellular DNA; Fg, fibrinogen; Fn, fibronectin; FnbA and FnbB, fibronectin-binding proteins A and B; *icaA* and *icaD*, intercellular adhesion A and D; MRSA, methicillin-resistant *Staphylococcus aureus*; P2 and P3, promoters 2 and 3; PIA, polysaccharide intercellular adhesion; and *sarA*, staphylococcal accessory regulator.

The key to the pathogenesis of *Staphylococcus aureus* (*S. aureus*) is its adhesion to host cells and components in the host extracellular matrix (Johannessen et al., 2012). The *S. aureus* genome encodes more than 20 adhesins that mediate initial biofilm cell attachment and intercellular adhesion during biofilm maturation, such as microbial surface component–recognizing adhesion matrix molecules (MSCRAMMs), including surface proteins such as fibronectin-binding proteins A and B (FnbA and FnbB), *clfA*, and *clfB* (Tristan et al., 2003) (Heilmann, 2011). MSCRAMMs have similar structures, with two adjacent IgG fold domains mediating their attachment to host extracellular matrix components such as collagen, fibrinogen, or fibronectin (Figure 8) (Foster et al., 2014). This binding ability is closely related to the pathogenicity of *Staphylococcus aureus* because its adhesion to the extracellular matrix or plasma proteins is a key step in the formation of biofilms. This finding is consistent with the increase in *fnbB* expression detected in our study. *clfA* is the major staphylococcal fibrinogen-binding protein, and its upregulation is responsible for the aggregation of *S. aureus* in the plasma, ultimately leading to arthritis and endocarditis (Foster, 2005).

Molecular studies have shown that during late phases of adherence, organisms first adhere to each other and then elaborate on a biofilm. This step is mediated by two biofilm components, PIA and the intracellular adhesion genes *icaA* and *icaD* (Figure 8) (Atshan et al., 2012). These genes are necessary for the synthesis of capsular polysaccharides/adhesion, which are regarded as the major components of MRSA biofilms (Chaieb et al., 2005). PIA is an important component of extracellular matrix–intercellular adhesion and is a key molecule that promotes bacterial cell adhesion (Mack et al., 1996; Vuong et al., 2004). Synthesis of the capsular polysaccharide is mediated by the *ica* operon. Upon activation of this operon, a polysaccharide intercellular adhesin is synthesized. This protein supports cell-to-cell bacterial contacts in a multilayered biofilm. The polysaccharide intercellular adhesin is composed of linear β-1, 6-linked glucosaminylglycans. They are synthesized *in vitro* from UDP-N-acetylglucosamine by the enzyme N-acetylglucosaminyltransferase, which is encoded by the *ica* locus, in particular by the *icaA* gene. The expression of *icaA* alone induces only low enzymatic activity, but coexpression of *icaA* with *icaD* leads to a significant increase in activity and is related to full phenotypic expression of the capsular polysaccharide (Gerke et al., 1998). Therefore, the inhibitory effect of AS on MRSA biofilms may be achieved by inhibiting bacterial adhesion.

The synthesis of virulence factors and other extracellular proteins of *S. aureus* is controlled by the QS system. In MRSA, the QS system is an *agr* system based on the autoinducer peptide (AIP) (Figure 8). The pre-AIP of *agr* is encoded by *agrD* and then processed into mature AIP by the membrane protein *agrB* and secreted out of the cell. *agrC* is a membrane receptor kinase that phosphorylates and activates *agrA* after binding to AIP. *agrA* regulates the expression of a series of downstream genes by binding promoter 2 (P2) and promoter 3 (P3). The RNAⅡ transcription frame starting at P2 includes the *agr* locus containing the *agrA*, *agrB*, *agrC,* and *agrD* genes, and the RNAⅢtranscription frame starting at P3 is an effector molecule of the *agr* system, encoding virulence factors such as α-hemolysin and δ-toxin (Asad and Opal, 2008). Researchers have speculated that AS inhibits the *agr* system of MRSA by interfering with the activity of *agrD*-encoding AIP, consistent with our RT-PCR results.

*sarA* is an important regulator of the QS system that positively regulates the production of biofilms and directly regulates the expression of several virulence factors in *S. aureus* (Zielinska et al., 2011; Sina et al., 2013). This protein is an important cause of the pathogenicity and recurrent infection of MRSA. The mechanism by which *sarA* positively regulates biofilms is its inhibition of extracellular enzymes (proteases and nucleases) (Abdelhady et al., 2014). The regulation of virulence factors by *sarA* is mediated by the binding of *sarA*-encoded proteins to peptides encoded in the upstream region, regulating the activation of P2 and P3 (Figure 8). P2 regulates the *agr* system through its own induction mechanism. P3 inhibits the production of biofilms and induces the expression of virulence factors by regulating the production of RNAⅢ (Blasi et al., 2016; Roy et al., 2018). In our experiment, the transcription of *sarA* was significantly downregulated after the AS intervention. This result is consistent with previous findings that AG (the parent compound of AS) significantly inhibits *sarA* activity. Therefore, AS exhibits antibiofilm and antivirulence activities by inhibiting *sarA* gene expression.

*cidA* is an important regulatory factor involved in the formation of *S. aureus* biofilms. The expression of *cidA* is related to bacterial adhesion and genomic DNA release and is continuously upregulated during biofilm formation (Figure 8) (Grande et al., 2014). The cid/LGR holin–antiholin system is related to the release of eDNA during cell lysis and planktonic growth, and it promotes adhesion and biofilm formation (Rice et al., 2007). eDNA, an important component of the extracellular polymer matrix, plays an important role in determining the stability and development of biofilms and is widely involved in the regulation of the formation of Gram-positive bacterial biofilms (Spoering and Gilmore, 2006). Activation of trichourea hydrolase contributes to the release of eDNA from biofilms (Bose et al., 2012). The protein encoded by *cidA* increases the activity of trichourea hydrolase and promotes bacterial separation from biofilms and dissemination to new infection sites (Ranjit et al., 2011). Researchers have suggested a potential role of *cidA* in the stage of biofilm adhesion or diffusion. Based on the results of the present study, the expression of *cidA* was significantly downregulated after the intervention of 25 mg/ml AS, suggesting that AS may inhibit the expression of eDNA regulated by *cidA* in the stage of biofilm adhesion or diffusion.

**FIGURE 9:**
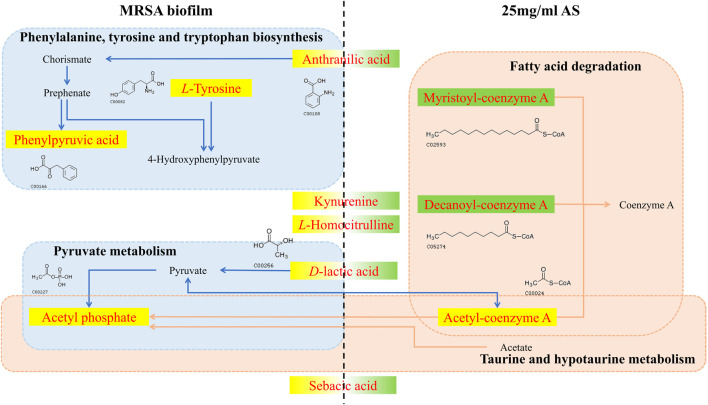
Changes in different metabolites and their metabolic pathways in MRSA biofilms treated with or without AS. The red font indicates the differentially altered metabolite in the pathway. The yellow background indicates that the level of the metabolite increased, and the green background indicates a decrease. The blue dashed boxes indicate pathways in which metabolic biomarkers of the MRSA biofilm are involved in the absence of the intervention. Pink dashed boxes indicate pathways in which pharmacodynamic metabolic markers of AS are involved.

In the process by which AS interferes with the formation of MRSA biofilms, AS significantly induced changes in the level of five metabolites, namely, anthranilic acid, KYN, *L*-homocitrulline, *D*-LA, and sebacic acid (Figure 9). Alterations in these metabolites might be responsible for the common formation of MRSA biofilms or therapeutic effects of drugs. Anthranilic acid is a key node in a complex pathway required for the synthesis of several important secondary metabolites in *Pseudomonas aeruginosa* (*P. aeruginosa*) (Vital-Lopez et al., 2015). Anthranilic acid facilitates the attachment of cells to the surface in the early stage of biofilm development. In addition, it affects the transcription of extracellular polymeric substance (EPS) operons. *P. aeruginosa* contains three major EPSs in the biofilm matrix: Psl, Pel, and alginate. Anthranilic acid treatment increases the transcription of the Psl operon by 85% (Vital-Lopez et al., 2015). Therefore, we speculate that AS inhibits the formation of biofilms by inhibiting anthranilic acid production in MRSA to affect cell adhesion and EPS production. KYN, the precursor of anthranilic acid, is critical for *P. aeruginosa* virulence. This bacterium utilizes multiple QS pathways to coordinate an arsenal of virulence factors. The kynurenine pathway (KP) has been implicated in the QS and virulence factor expression (Kasper et al., 2016). The downregulation of KYN levels may be related to the effect of the AS intervention on the transcription of QS-related genes (*agrD* and *sarA*). *D*-LA, a natural organic acid, is an important carbon and energy source for pathogens and commensal bacteria in mammalian hosts (Jiang et al., 2014; Gillis et al., 2018; Lin et al., 2018). In addition, *D*-LA can be used as an electron donor for aerobic respiration in the upper, oxic portion of the biofilm (Lin et al., 2018). *P. aeruginosa* is the most common cause of chronic, biofilm-based lung infections in patients with cystic fibrosis (CF). The lung environment of patients with CF might nevertheless contain subregions where lactate is a major source of carbon and energy (Lin et al., 2018). Furthermore, PA14 has the potential to utilize *D*-LA during growth in colony biofilms (Lin et al., 2018). Thus, the increased level of *D*-LA may play a positive role in promoting biofilm formation by providing an energy source. The downregulation of *D*-LA may contribute to AS-induced decreases in the carbon and energy sources needed for the growth of the MRSA biofilm and subsequent damage.

Phenylpyruvic acid, *D*-2-aminobutyric acid, and *L*-tyrosine are characteristic metabolites involved in the formation of MRSA biofilms. Treatment with 25 mg/ml AS did not alter the levels of these metabolites. Phenylpyruvic acid is formed by the decomposition of phenylalanine into *L*-tyrosine (Wang et al., 2017). Phenylpyruvic acid is involved in the metabolism of amino acids during the formation of MRSA biofilms. *D*-2-Aminobutyric acid, an unnatural amino acid, enhances the activity of phosphatase, which can hydrolyze phosphate ester and polyphosphoric acid compounds. *D*-2-Aminobutyric acid levels decrease in the process of biofilm formation, ensuring the formation of phosphoteichoic acid as a cell wall component. By performing a metabolite pathway analysis, these metabolites were found to be involved in the biosynthesis of phenylalanine, tyrosine, and tryptophan, as well as in taurine and hypotaurine metabolism. These two metabolic pathways may significantly affect the formation of MRSA biofilms. D-Urobilin, decanoyl-coenzyme A, myristoyl-coenzyme A, and acetyl-coenzyme A are biomarkers reflecting the therapeutic effect of AS. The metabolic pathway analysis showed that glyoxylate and dicarboxylate metabolism and fatty acid degradation were the most significant pathways of terminal metabolites reflecting the therapeutic effect of AS.

## Conclusions

**FIGURE 10:**
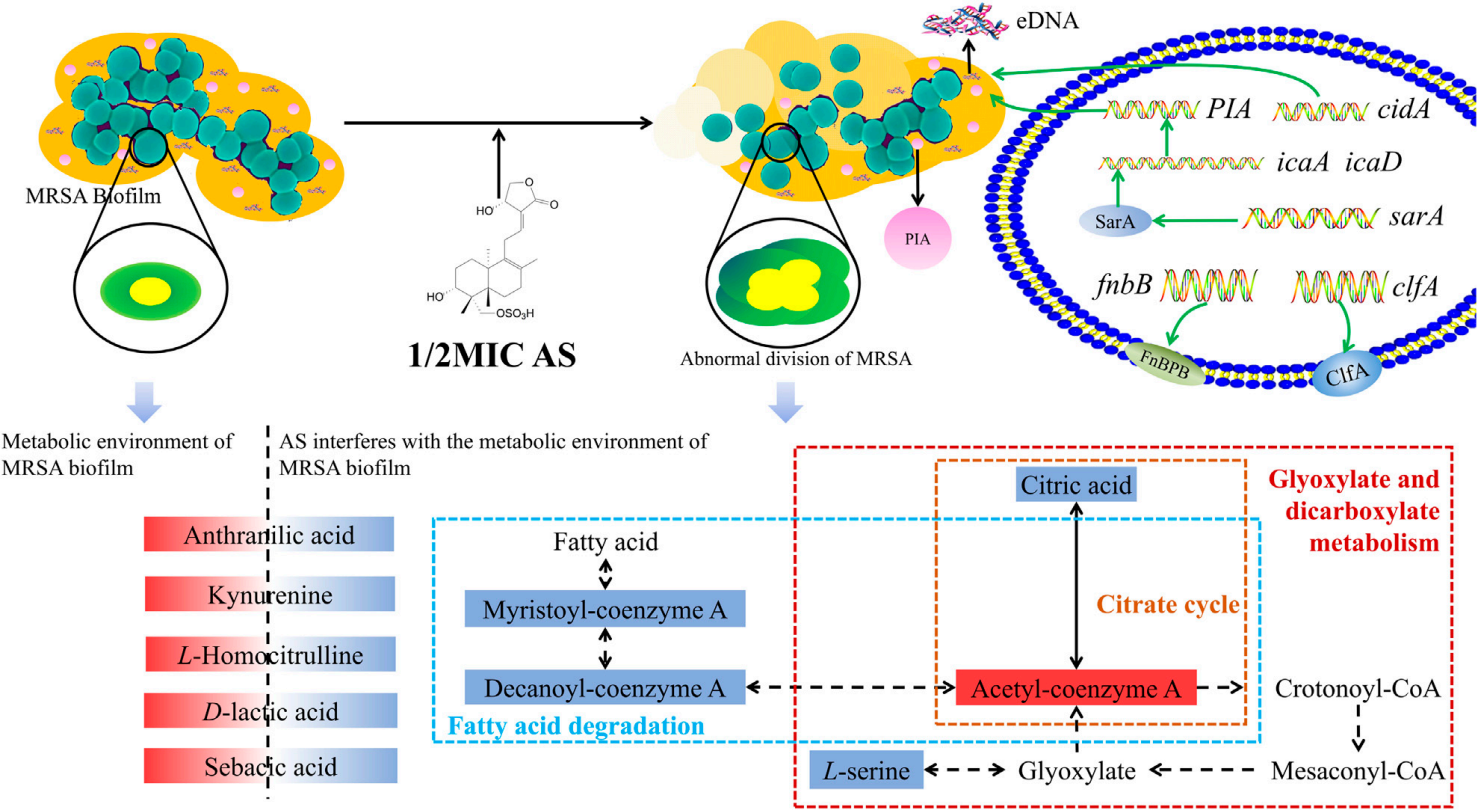
Overview of the technical analyses described in the article. eDNA, extracellular DNA; PIA, polysaccharide intercellular adhesion; *icaA*, intercellular adhesion A; *icaD*, intercellular adhesion D; and FnbB, fibronectin-binding protein B. The red solid box represents upregulated metabolite levels, and the blue box represents the downregulated levels. The blue dashed box represents the fatty acid degradation pathway, the yellow dashed box represents the citrate cycle pathway, and the red box represents glyoxylate and dicarboxylate metabolism.

MRSA biofilms are one of the causes of antibiotic resistance, and their formation can be inhibited by AS. AS not only inhibits the survival of MRSA in the biofilms but also inhibits the proliferation of MRSA by interfering with cell division. Its mechanism is mediated by downregulating the transcription of genes related to the QS system, bacterial adhesion, and eDNA release and interfering with the amino acid, fatty acid, taurine, and secondary taurine metabolism in MRSA (Figure 10). This study provides a theoretical foundation for the application of AS as an anti-MRSA biofilm agent.

## Data Availability

The datasets presented in this study can be found in online repositories. The names of the repository/repositories and accession number(s) can be found in the article.
